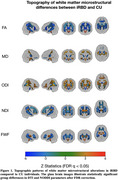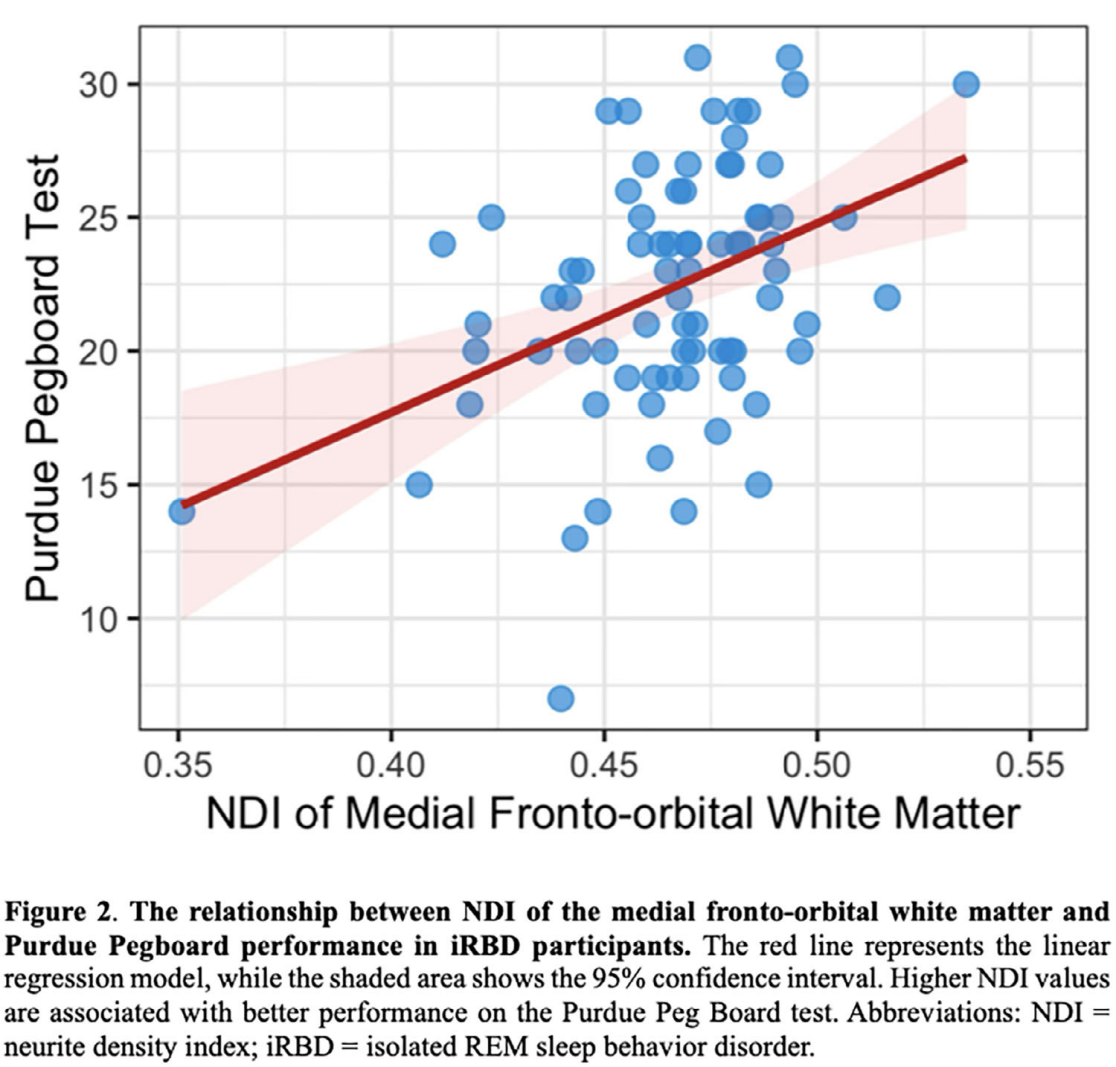# NODDI reveals white matter microstructural changes in isolated REM sleep behavior disorder

**DOI:** 10.1002/alz70856_106325

**Published:** 2026-01-10

**Authors:** Elijah Mak, Scott A. Przybelski, Robert I. Reid, Angela J Fought, Christopher G Schwarz, Prashanthi Vemuri, Clifford R. Jack, Yuzheng Nie, Hernis De La Cruz, Alon Y Avidan, Donald Bliwise, Meghan Campbell, Susan Criswell, Albert A Davis, Kevin Duff, Kaylena Ehgoetz Martens, Jonathan Elliot, Tanis J Ferman, Julie A. Fields, Leah K. Forsberg, Jean‐François Gagnon, Ziv Gan‐Or, Michael J Howell, Michele Hu, Xiaoping Hu, Daniel Huddleston, Paul T Kotzbauer, Jason Langley, Miranda Lim, Jessica Locke, Val J Lowe, Stuart J McCarter, Jennifer S McLeland, Mitchell Miglis, Emmanuel Mignot, Toji Miyagawa, Lee Neilson, Kendall James Nichols, Amelie Pelletier, Owen A. Ross, Carlos H Schenck, Singer Wolfgang, Erik K St. Louis, Lynn Marie Trotti, Aleksandar Videnovic, Chengjie Xiong, Ronald B Postuma, Yo‐El Ju, Brad F Boeve, Kejal Kantarci, the NAPS Consortium

**Affiliations:** ^1^ Mayo Clinic, Rochester, MN, USA; ^2^ Department of Quantitative Health Sciences, Mayo Clinic, Rochester, MN, USA; ^3^ Department of Radiology, Mayo Clinic, Rochester, MN, USA; ^4^ Washington University, Washington, WA, USA; ^5^ University of California at Los Angeles, Los Angeles, CA, USA; ^6^ Emory University School of Medicine, Atlanta, GA, USA; ^7^ Washington University in St. Louis, St. Louis, MO, USA; ^8^ Barrow Neurological Institute, Phoenix, AZ, USA; ^9^ Oregon Health & Science University, Portland, OR, USA; ^10^ University of Waterloo, Waterloo, ON, Canada; ^11^ University of Oregon, Portland, OR, USA; ^12^ Department of Psychiatry and Psychology, Mayo Clinic, Jacksonville, FL, USA; ^13^ Department of Psychiatry and Psychology, Mayo Clinic, Rochester, MN, USA; ^14^ Department of Neurology, Mayo Clinic, Rochester, MN, USA; ^15^ Université du Québec à Montréal, Montreal, QC, Canada; ^16^ The Neuro (Montreal Neurological Institute), Montreal, QC, Canada; ^17^ University of Minnesota, Minneapolis, MN, USA; ^18^ Nuffield Department of Clinical Neurosciences, University of Oxford, Oxford, United Kingdom; ^19^ University of California, Riverside, Riverside, CA, USA; ^20^ Washington University School of Medicine, Washington, WA, USA; ^21^ Oregon Health and Science University, Portland, OR, USA; ^22^ Washington University School of Medicine, St Louis, MO, USA; ^23^ Stanford University, Stanford, CA, USA; ^24^ Oregon Health & Science University, Oregon, OR, USA; ^25^ Emory University, Atlanta, GA, USA; ^26^ McGill University, Montreal, QC, Canada; ^27^ Mayo Clinic, Jacksonville, FL, USA; ^28^ Massachusetts General Hospital/Harvard, Boston, MA, USA; ^29^ Washington University School of Medicine in St. Louis, St. Louis, MO, USA; ^30^ Centre for Advanced Research in Sleep Medicine, Hôpital du Sacré‐Cœur de Montréal, Montreal, QC, Canada; ^31^ Washington University in St. Louis, St Louis, MT, USA

## Abstract

**Background:**

Isolated REM sleep behavior disorder (iRBD) is a parasomnia that reflects an evolving α‐synucleinopathy disorder, providing an opportunity to study early pathological changes. While diffusion tensor imaging (DTI) studies have shown white matter changes in iRBD, Neurite Orientation Dispersion and Density Imaging (NODDI) may offer better biological specificity in characterizing microstructural alterations through measures of Neurite Density Index (NDI), Orientation Dispersion Index (ODI), and Free Water Fraction (FWF).

**Method:**

We included 77 participants with polysomnography‐confirmed iRBD from the North American Prodromal Synucleinopathy (NAPS) Consortium and 154 age‐ and sex‐matched cognitively unimpaired controls from the Mayo Clinic Study of Aging. White matter microstructure was evaluated using standardized multi‐shell diffusion on 3T MRI, quantifying DTI metrics (fractional anisotropy, FA; and mean diffusivity, MD) and NODDI parameters across bilateral white matter tracts defined by the JHU “Eve” WM atlas. Group differences were assessed using conditional logistic regression, with correlations to motor performance evaluated using the Purdue Pegboard and Alternating Finger Tapping tests.

**Result:**

Compared to controls, iRBD participants demonstrated widespread and bidirectional white matter changes across major white matter pathways (see Figure 1 for glass brain visualizations). While predominantly showing decreases, FA exhibited some increases, particularly in the corticospinal tract. MD showed a largely opposite pattern with predominantly increased values across tracts. NODDI metrics revealed complex bidirectional patterns: ODI was broadly increased across multiple tracts with focal decreases, while NDI showed a pattern of predominantly decreased values alongside localized increases. FWF demonstrated a mixed pattern with predominant decreases across most tracts. In addition, both DTI and NODDI metrics showed extensive, moderate correlations with the Purdue Pegboard Test and Alternating Finger Tapping performance (*T* = 2.0, *p* < 0.05, Figure 2).

**Conclusion:**

Our study highlighted widespread and complex bidirectional white matter microstructural alterations in individuals with iRBD, demonstrating significant correlations with dexterity and motor performance even during the prodromal stage. These results suggest that extensive white matter abnormalities occur early in prodromal α‐synucleinopathies and highlight the value of advanced diffusion imaging techniques in characterizing iRBD and its potential prediction of phenoconversion to overt neurodegenerative disorders.